# Clearing the air: a systematic review on leadership challenges with sustainable inhaler prescribing

**DOI:** 10.1136/leader-2025-001257

**Published:** 2025-08-06

**Authors:** Sten Kajitani, Anthony Goodings, Yasmina Richa, Asis A Babun, Allison Chhor, Roberto Velasco, Juan Trujillo

**Affiliations:** 1 University College Cork, Cork, Ireland; 2 Memorial University of Newfoundland, St. John’s, Newfoundland and Labrador, Canada; 3 University College Cork College of Medicine and Health, Cork, Ireland; 4 Rocky Vista University College of Osteopathic Medicine, Parker, Colorado, USA; 5 University of Ottawa, Ottawa, Ontario, Canada; 6 Parc Taulí Hospital Universitari, Sabadell, Spain; 7 Cork University Hospital, Cork, Ireland

**Keywords:** sustainability, health system, improvement

## Abstract

**Background:**

The environmental impact of inhalers, particularly pressurised metered dose inhalers with high global warming potential, poses significant challenges in the context of planetary health. Although dry powder inhalers (DPIs) offer a more sustainable alternative, entrenched prescribing practices prevail. This systematic review evaluates patient and physician perspectives on inhaler environmental impacts and examines barriers and opportunities for leadership in adopting sustainable practices.

**Methods:**

Following Preferred Reporting Items for Systematic Reviews and Meta-Analyses guidelines, a comprehensive literature search was performed from inception to 12 June 2024, across Medline via EBSCO, EMBASE via Elsevier and Web of Science. Four studies were included, surveying 433 participants. Data extraction and risk-of-bias assessment were conducted using a standardised form and the Newcastle-Ottawa Scale.

**Results:**

Findings indicate that while both patients and providers express environmental concerns, limited awareness and entrenched clinical practices hamper the transition to DPIs. Leadership insights reveal that a fragmented sense of responsibility, insufficient training and low confidence in discussing environmental impacts are significant barriers. However, targeted education and interprofessional collaboration have been shown to increase the willingness to adopt sustainable inhaler practices.

**Conclusions:**

The results underscore the need for leadership in healthcare to champion sustainable prescribing. Empowering clinicians through education, clear clinical guidelines and eco-ethical leadership initiatives is essential. Health leaders have the opportunity to transform practice by integrating environmental considerations into routine care, ultimately advancing planetary health.

**The PROSPERO registration number:**

CRD42024552555

**WHAT IS ALREADY KNOWN ON THIS TOPIC**

- Pressurised metered dose inhalers are known to have a high environmental impact due to their propellant emissions, while dry powder inhalers (DPIs) offer a more sustainable alternative. Despite this, entrenched prescribing habits, limited environmental awareness and knowledge gaps among healthcare professionals have hindered the adoption of DPIs.

**WHAT THIS STUDY ADDS**

- This systematic review highlights that fragmented leadership, low provider confidence and inadequate interprofessional collaboration are significant barriers to sustainable inhaler prescribing. It also supports that targeted educational interventions and eco-ethical leadership initiatives may increase the willingness to adopt environmentally friendly practices.

**HOW THIS STUDY MIGHT AFFECT RESEARCH, PRACTICE OR POLICY**

- The findings call for a reorientation of clinical leadership to integrate planetary health principles into everyday practice. They underscore the need for policy changes, enhanced training programmes and interprofessional strategies to promote sustainable inhaler use, potentially transforming respiratory care and advancing environmental stewardship in healthcare.

## Introduction

 The escalating threat of climate change necessitates urgent action across all sectors, including the healthcare sector, as it significantly contributes to global greenhouse gas emissions.^1 2^ Among various medical practices, the use of inhalers for managing asthma and other respiratory conditions has been identified as having a negative environmental impact.^3^

Pressurised metered dose inhalers (pMDIs), widely prescribed for their effectiveness and ease of use, employ hydrofluoroalkane (HFA) or chlorofluorocarbon (CFC) propellants, which have approximately 3200 or 10 900 times the warming potential of carbon dioxide, respectively.^4^,^6^ In contrast, dry powder inhalers (DPIs) do not require propellants, presenting a more environmentally sustainable alternative.^7 8^

Of note, in 1998, only 13% of 201 patients knew that pMDIs contained CFCs, and on learning that pMDIs contained CFCs, 89% of respondents believed that pMDIs should be made CFC-free.^9^ Patients who knew that pMDIs contained CFCs were more inclined to switch to CFC-free inhalers (89%) compared with those who did not (50%), a relationship confirmed by multiple logistic regression analysis (adjusted OR=2.83, 95% CI (2.31 to 3.35)).^9^ When offered a range of alternatives, 55% of respondents expressed willingness to switch to CFC-free pMDI formulations, while 11% preferred DPIs and 34% were unsure.^9^

The phase out of CFCs was part of a major global effort to protect the ozone layer, as outlined by the Montreal Protocol in 1987.^10^ This international treaty was widely covered by the media and government worldwide, highlighting the importance and rationale behind the transition from CFC to HFA inhalers as well as reassurance on the safety and efficacy of HFA inhalers.^11 12^ In contrast, knowledge dissemination about the environmental impact of HFA pMDIs compared with DPIs remains limited to specific environmental and medical communities. Patients are, therefore, dependent on their healthcare providers to learn about the nuances of pMDIs vs DPIs and their environmental impacts.^9 13^

While the shift from CFC to HFA inhalers was well received and effectively prevented further ozone depletion, it did not lead to as significant of a reduction in carbon emissions as initially expected.^6^ HFAs, while less potent of a greenhouse gas compared with CFCs, still carry up to 3220 times greater global warming potential compared with carbon dioxide.^6^ Further efforts to reduce the overall carbon footprint associated with inhalers must involve patient and provider education and strong institutional and public support, as demonstrated by the successful transition from CFC to HFAs.^10^

Despite the environmental benefits of DPIs, their adoption in clinical practice remains inconsistent.^14 15^ While some factors limiting DPI prescription are well known, such as the inability to use current DPI inhalers by those with unsuitable inspiratory flow or coordination, such as preschool children, patients with cognitive or motor limitations and patients with COPD. Several other factors influencing the prescribing behaviours of physicians, and the awareness and attitudes of patients towards the environmental impact of inhalers, are not entirely understood.^16^ Addressing this gap is critical for developing strategies to promote sustainable inhaler prescribing practices in respiratory care.

In the context of growing environmental challenges, this systematic review explores the leadership opportunities and obstacles in advancing sustainable change within healthcare. Healthcare leaders, including physicians, nurses, allied health professionals, hospital administrators and executives, and healthcare policymakers, are called on to implement evidence-based strategies that reduce environmental impacts while maintaining high-quality patient care. In addition to modifying the formulations of prescribed medication, appropriate diagnosis and adequate patient follow-up are essential to optimise treatment. Such measures will reduce both financially and environmental impact by limiting unnecessary medication use and reducing hospitalisations.^17 18^ By systematically examining the existing literature, the review evaluates how environmental knowledge influences prescribing patterns between DPIs and pMDIs. It also identifies key barriers and facilitators to the adoption of more environmentally sustainable inhaler options. The insights gained are intended to inform the development of targeted educational initiatives and clinical guidelines, thereby fostering environmentally responsible prescribing practices that align with broader healthcare sustainability goals.

## Methods

### Study design

This systematic review was conducted following the Preferred Reporting Items for Systematic Reviews and Meta-Analyses guidelines. The review aimed to evaluate the attitudes and awareness of patients and physicians regarding the environmental impacts of inhalers, with a focus on identifying leadership-related barriers to sustainable prescribing, particularly regarding the choice between pMDIs and DPIs. The PROSPERO registration number is CRD42024552555.

### Search strategy

A comprehensive literature search was performed from inception to 12 June 2024, across three electronic databases: Medline via EBSCO, EMBASE via Elsevier and Web of Science. Additionally, a literature search was conducted by reviewing Google Scholar search results. The search strategy incorporated keywords and Medical Subject Headings terms based on the Population, Exposure, Comparison, Outcome framework. Detailed search strategies are provided in online supplemental appendix 1.

- **Population:** “Physicians”, “General Practitioners”, “Pulmonologist”, “Doctors”, “Patients”
- **Exposure**: “Metered Dose Inhalers”, “Propellant inhalers”, “Pressurized inhalers”, “Aerosol inhalers”, “CFC inhalers”, “HFA inhalers”
- **Comparison:** “Dry Powder Inhalers”, “Powder inhalers”, “Breath-activated inhalers”, “Capsule inhalers”
- **Outcomes**: “Prescription Patterns and Patient Satisfaction”, “Recycling Practices”, “Awareness and Willingness for Change”, “Cost Considerations”, “Subjective Experience Factors”, “Level of Prescriber Responsibility”, and “Prescriber Knowledge and Confidence”

The search strings combined these terms using Boolean operators (AND, OR) to ensure a thorough search of the relevant literature (online supplemental appendix 1).

### Study selection

Two authors (YR and AAB) independently conducted the initial screening and full-text review based on the eligibility criteria, utilising Rayyan and Zotero for organisation and analysis (online supplemental appendix 2).^19 20^ Any conflicts were resolved by a third author (SK or AG).

### Data extraction

A standardised data extraction form was used to collect relevant information from each included study. Two independent reviewers were involved in data collection (SK and AAB). Extracted data included study characteristics, author(s), year of publication, country, study design, sample size, population characteristics, exposure details, type of inhalers (MDI vs DPI), prescribing practices, attitudes towards environmental sustainability, awareness of environmental impact and insights related to leadership challenges and facilitators to the adoption of DPIs.

### Quality assessment

The quality of the included studies was assessed using the Newcastle-Ottawa Scale (NOS) by two independent reviewers (SK and AB).

### Data synthesis

Weighted averages of outcomes were determined based on the size of each study population. Given the heterogeneity of the included studies, a meta-analysis was not possible; therefore, a narrative review was conducted.

## Results

**Figure 1:**
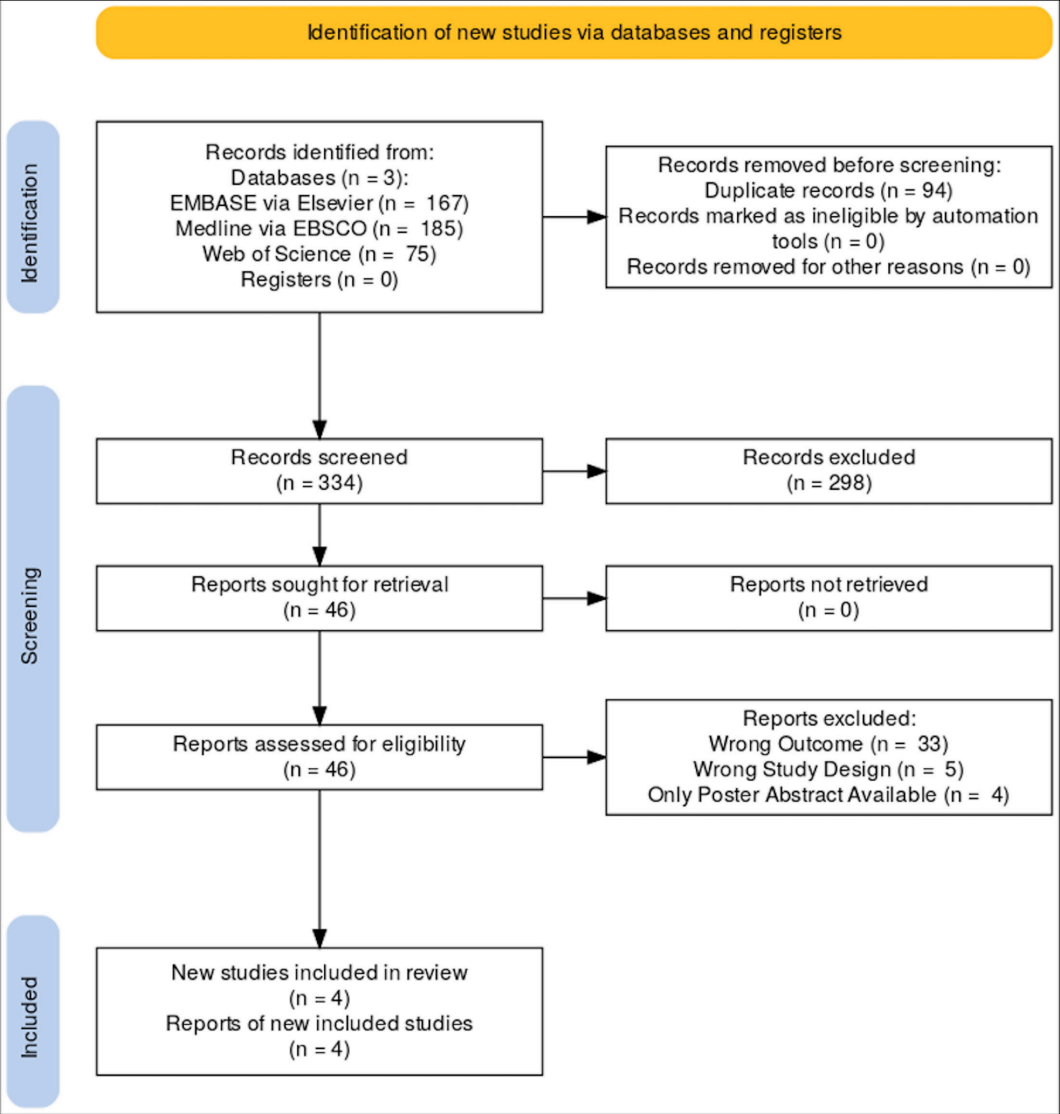
PRISMA flowchart. Illustrates the systematic process of identifying, screening and including studies in our review on the awareness of environmental impact and attitudes towards sustainable inhaler prescribing. Initially, we identified 334 records through comprehensive database searches, which included 167 records from EMBASE via Elsevier, 185 from Medline via EBSCO and 75 from Web of Science. After removing 94 duplicate records, we proceeded with 240 records for screening. During the screening phase, we excluded 298 records based on their titles and abstracts. Consequently, we sought retrieval of 46 full-text reports for a detailed eligibility assessment (see online supplemental appendix 2). Among these, we excluded 42 reports due to various reasons: 33 reports had wrong outcomes, 5 had incorrect study designs and 4 were only available as poster abstracts. Ultimately, this rigorous process led to the inclusion of four new studies in the review. The PRISMA flowchart effectively demonstrates the meticulous approach taken to ensure a thorough and unbiased selection of relevant studies. PRISMA, Preferred Reporting Items for Systematic Reviews and Meta-Analyses.

Following a comprehensive search, a total of 46 studies were retrieved for full-text assessment, of which four met the inclusion criteria and were included in this systematic review (figure 1).^9 13 16 21^ Three of these studies were completed in English-speaking countries (Canada, New Zealand, United Kingdom) and one was completed in Singapore. These four studies collectively surveyed 433 participants (patients and healthcare professionals). One of the four studies included focused on the transition from CFC to HFA propellants, while this study is somewhat of an outlier, it provides valuable insight on patient perspectives and system-level changes in the inhaler industry. This may be of great use when working towards system-level solutions and the role of patient and professional advocacy. Table 1 summarises the key characteristics of each study, while table 2 outlines the results of the NOS risk-of-bias assessment, and author affiliations and bias assessment tables are more comprehensively described in online supplemental appendix 3, indicating that all included studies achieved high quality. Below is a narrative synthesis of the main findings from the included studies, organised under key outcomes related to inhaler use and environmental sustainability.

**Table 1:** Summary of included studies

Author	Year	Journal	Included subjects (characteristics and N)	Main result
Goh *et al*	1998	Annals of Tropical Paediatrics	Parents of paediatric asthma patients in Singapore, N=201	After learning about CFCs, 89% of patients supported CFC-free pMDIs; 55% would switch, 11% preferred DPIs, and 34% were unsure. Effectiveness (97%), safety (95%) and doctor’s recommendation (94%) were prioritised over environmental friendliness (55%), canister attractiveness (45%) and manufacturer (6%).
Walpole *et al*	2021	Future Healthcare Journal	Hospital prescribers in an NHS trust, N=102	A 4-week survey of 102 prescribers found only 2% answered environmental impact questions correctly, with many unsure about the carbon footprint of inhalers. Nearly half felt they could discuss lower-carbon options with support. Regarding recycling, 55.9% were unaware inhalers could be recycled at pharmacies.
Woodall *et al*	2023	New Zealand Medical Journal	Patients and practitioners in New Zealand, N=69 (53 patients, 16 practitioners)	Among 53 patients (64% pMDI, 53% DPI, 15% SMI, 32% multiple inhalers), 89% rated symptom relief, 70% ease of use and 68% environmental impact as important for changing inhalers, with 83% viewing global warming as crucial. For 16 practitioners, symptom relief and ease of use were key, with environmental impact importance rising from 25% to 75% after information was provided.
Quantz *et al*	2023	Canadian Pharmacists Journal	Residents of Fraser Health Authority in British Columbia, N=61	61 completed the survey, with 70% older than 51 years; 61% used pMDIs and 20% returned empty inhalers to pharmacies. While only 21% were aware of inhalers' carbon footprints, 59% valued low-carbon options, needing more information (35%), prescriber advice (29%) and pharmacist support (26%) to switch, with cost and convenience being major barriers.

This table provides an overview of four key studies examining the awareness and attitudes of patients and physicians towards the environmental impact of inhalers.

CFC, chlorofluorocarbon; DPI, dry powder inhaler; NHS, National Health Service; pMDI, pressurised metered dose inhaler; SMI, soft mist inhaler.

**Table 2:** Risk of bias assessment Newcastle-Ottawa Scale summary

Study	Selection	Comparability	Outcome
Woodall *et al*	★★★★	★★	★
Walpole *et al*	★★★★	★★	★
Goh *et al*	★★★★	★★	★
Quantz *et al*	★★★★	★★	★

This table presents the risk of bias assessment for the included studies, evaluated using the Newcastle-Ottawa Scale. The risk of bias assessment was conducted by two independent reviewers.

### Prescription patterns and patient satisfaction

Across studies, pMDIs were the most commonly prescribed devices, used by approximately 71% of patients, compared with 26% using DPIs (N=315).^9 13 16^ One study involving both patients and practitioners (N=53) reported that satisfaction was higher among users of DPIs (89%) than among pMDI users (75%), although no p values were provided.^16^ Similarly, despite DPIs being perceived as more environmentally friendly, studies highlighted that many prescribers and patients persisted in favouring pMDIs due to concerns about familiarity, perceived effectiveness and ease of use.^9 13 16^

### Recycling practices

Only 20% of patients (n=61) reported returning their empty inhalers to a pharmacy for proper disposal, with the majority discarding inhalers in household waste.^13^ Among 102 prescribers surveyed, only 13 (12.7%) claimed to routinely discuss recycling with patients, and over half were unaware of the possibility that inhalers could be recycled at pharmacies (n=57).^21^ This lack of awareness on both patient and provider sides appears to contribute to poor recycling rates.

### Awareness and willingness for change

#### Patients

Three of the four studies reported on patient awareness regarding the environmental impact of inhalers, with approximately 49% of patients (n=153) demonstrating environmental awareness (N=315).^9 13 16^ In these same studies, 57% of 315 (n=183) patients reported that environmental friendliness/environmental impact was an important factor influencing inhaler choice, and 58% of 315 (n=175) patients expressed willingness to switch to sustainable inhalers.^9 13 16^

#### Practitioners

Interestingly, one study specifically documented that before receiving information on environmental impacts, 12 out of 16 practitioners (75%) believed global warming to be ‘very’ or ‘extremely’ important, but only 10 practitioners were aware of the differences in global warming potential between pMDIs and DPIs.^16^ After being informed about the environmental impact of different inhalers, practitioners significantly increased their intention to consider the environment when prescribing, from 25% before receiving information to 75% afterward (p=0.029).^16^

### Cost considerations

Cost emerged as a pivotal factor influencing both patient and prescriber decisions. In one survey, 49% of 53 patients (n=26) reported that cost to the healthcare system was an important factor. While another study reported that 93% of 201 patients were willing to change if the new inhalers were cost equivalent to their current ones.^9^ This willingness decreased to 68% if the cost increased by up to one-and-a-half times, 44% if the cost doubled and 31% if the cost tripled.^9^

### Subjective experience factors

Across studies, effectiveness, safety and ease of use consistently ranked as top considerations for choosing an inhaler. Between two studies, 95% of 254 patients (n=242) reporting that effectiveness/symptom relief was an important factor.^9 16^ Furthermore, 95% of 201 (n=191) patients reported that safety was an important factor.^9^ Additionally, 70% of 53 patients reported that ease of use was an important factor (n=37).^16^

Another subset placed value on features such as ease of use, taste and canister/packaging design. Notably, 45% of 201 patients (n=90) stated that the attractiveness of the canister and packaging was an important factor.^9^ 6% of 201 patients (n=12) stated that the manufacturing pharmaceutical company was an important factor.^9^

### Level of prescriber responsibility

Importantly, 35% of 61 patients (n=21) identified the need for more information as the most significant support for switching to a low-carbon inhaler.^13^ This is arguably the responsibility of the prescriber, as 77% of 262 patients reported that doctor’s recommendation/advice from prescriber was an important factor (n=207).^9 13^ In practice, only 77% of 61 patients reported receiving some level of inhaler training in the past year, primarily from pharmacists, respiratory therapists or doctors.^13^ However, 16% did not receive any training when given a new inhaler.^13^

This observation might be due to the fact that when surveyed about their roles, prescribers showed varying degrees of responsibility for initiating or switching inhalers. Respiratory physicians were the most likely to accept responsibility, with 83.3% of 12 respiratory physicians stating they were responsible for initiating inhalers, and 100% claiming responsibility for changing inhalers.^21^ However, only 37.5% of eight other consultant doctors felt that they were responsible for both initiating and changing inhalers, and only 25.0% of 21 junior doctors felt responsibility for initiating inhalers, and only 21.9% felt responsible for changing them.^21^ Foundation doctors in the United Kingdom felt the lowest initiation responsibility at 19.0% of 21, with 23.8% responsible for changing inhalers.^21^ It is also interesting to mention that 26% of 61 patients (n=16) identified support from a pharmacist as an important factor.^13^ However, only 13.6% of 22 pharmacists felt responsible for initiating and 36.4% for changing inhalers.^21^ The fragmented sense of responsibility and lack of leadership potentially explains why patients did not receive uniform counselling on environmentally sustainable inhaler options.

### Prescriber knowledge and confidence

A notable knowledge gap was observed among prescribers, with an average correct response rate of only ~30% on surveys testing 102 prescribers’ understanding of inhalers’ relative environmental impacts.^21^ Only 2 out of 102 respondents (2.0%) answered all three questions correctly, while 13 out of 102 (12.7%) incorrectly thought that DPIs had a higher carbon footprint than pMDIs.^21^

This lack of knowledge translated to prescribers’ confidence in discussing lower carbon inhaler choices with their patients, with only 8.8% of 102 prescribers, surveyed already feeling prepared to counsel patients about lower carbon inhaler choices.^21^ An additional 41.2% did not feel confident discussing this with patients.^21^ However, 46.1% of prescribers felt they could do so with additional support such as training, senior guidance or a patient decision tool.^21^

## Discussion

This systematic review highlights the multifaceted leadership challenges associated with implementing sustainable inhaler prescribing in clinical practice and underscores a notable gap in both patient and physician awareness of inhalers’ environmental impacts. Although previous transitions in inhaler propellants—for instance, from CFCs to HFAs—were relatively successful due to widespread media coverage and government backing, our findings suggest that current efforts to promote DPIs as a lower carbon alternative have yet to achieve comparable momentum.^4^,^6^ Greater awareness and advocacy, particularly by fostering patient and professional leaders, may lead to greater pressure on regulatory bodies to mandate changes in prescribing guidelines. Across the four included studies, pMDIs remained the most commonly prescribed and used inhaler type, despite many prescribers and patients displaying willingness to switch to DPIs when adequately informed of their environmental benefits.^9 13 16 21^ It is also important to address the geographic homogeneity of these studies as they are based in Commonwealth and North American healthcare settings where a persistent cultural prescribing pattern that favours pMDIs exists relative to other regions in the world including Mainland Europe and Scandinavian countries.^15^

A critical insight from our review is that the knowledge gap regarding inhaler-associated emissions persists among both patients and healthcare professionals. While roughly half of patients were aware of the environmental implications of pMDIs and HFAs, even fewer reported confident understanding of recycling practices, resulting in most empty inhalers being disposed of with household waste.^13 21^ Physicians were similarly underinformed; in one study, fewer than one-third of prescribers correctly understood the relative carbon footprints of different inhaler types.^21^ This deficiency in provider knowledge was consistently accompanied by low self-reported confidence in counselling patients about switching to or initiating sustainable alternatives.^21^ However, study participants who received targeted education about the global warming potential of inhalers displayed a marked increase in their inclination to prescribe or use DPIs.^16^ Such findings underscore the importance of structured educational programmes for providers and patients alike.^21 22^ Leaders in respiratory care and pharmacy can champion continuous education to close the knowledge gap. Tailored interventions through decision aids, online modules or dedicated respiratory clinics, could address misconceptions about the efficacy of DPIs and empower healthcare professionals to facilitate more environmentally responsible prescribing practices.^16 21^

The concept of eco‐ethical leadership, which emphasises ethical responsibility towards the environment alongside clinical excellence, is particularly relevant here.^23^ Eco‐ethical leaders advocate for practices that minimise harm to both patients and the planet. By incorporating the principles of eco‐ethical leadership, healthcare leaders can drive change by modelling sustainable behaviours, fostering interprofessional collaboration and innovating solutions.^23^ Similarly, the planetary healthcare framework, which situates health systems within the broader context of environmental sustainability, calls for integrating ecological considerations into everyday clinical decision-making.^24^ Both frameworks support the idea that leadership is not just about clinical outcomes but also about stewarding resources responsibly to ensure long-term planetary health.

Financial factors also substantially influence prescription patterns and patient decisions.^9 16^ While most patients express willingness to transition to a more expensive DPI if it offers equivalent or superior environmental benefits, this willingness diminishes as the cost differential increases.^9^ However, the cost of DPIs relative to pMDIs has been found in some studies to be cost-effective over time, mostly due to reduced public health expenditures.^25^ This suggests that initiatives undertaken by several countries to subsidise the cost of prescription medication are well placed. Implementation of universal prescription drug insurance is variable between and within countries, for instance, within Canada, the province of Québec has had universal prescription drug insurance (under the Régime général d'assurance médicaments) since 1997, whereas the rest of Canada has only recently begun to implement a similar programme (under bill C-64) in 2024. Moreover, the availability of DPIs can vary significantly across countries, impacting respective patients’ ability to access these alternatives.^15^

Hence, leaders in medicine could partner with governmental and industry stakeholders to ensure that sustainable inhaler options remain both clinically effective and affordable to broad patient populations. While cost barriers can be mitigated through pricing regulations or formulary changes that favour DPIs, these strategies must be paired with robust communication campaigns outlining the long-term benefits—both environmental and potentially economic—of adopting lower-carbon inhalers.^26^

A major source of DPI adoption is the most recent iteration of the Global Initiative for Asthma (GINA) guidelines. These recommend single maintainer and reliever therapy using budesonide-formoterol. This has empowered clinicians to switch patients of off suboptimal therapies such as salbutamol pMDI mono therapy or dual pMDI inhaler therapy that uses an ICS such as fluticasone or beclomethasone.^27 28^

A further complicating factor is the fragmentation of responsibility among clinicians for initiating, switching or discussing inhaler options with patients. Respiratory specialists are more likely to take leadership for these decisions but generalists and junior doctors often feel less empowered or knowledgeable, resulting in inconsistent messaging to patients.^13 21^ Pharmacists, who frequently provide inhaler training, similarly reported limited confidence in discussing environmental concerns or suggesting viable alternatives.^13 21^ This diffusion of responsibility can undermine any single intervention’s effectiveness and emphasise the need for institution-wide or national-level policies that clearly delineate roles.^9^ Standardised protocols, embedded in electronic prescribing systems or clinical practice guidelines, could bolster confidence, ensuring that patients receive uniform advice about available sustainable inhaler options. By drawing on the planetary healthcare framework, institutions can develop integrated strategies that promote a shared vision of sustainable care.^24^

Some patients, especially young children and the elderly, may find DPIs challenging to use due to the requirement for adequate inspiratory pressure.^29^,^31^ Perhaps developing and promoting DPIs that are easier to use for all patient groups, including children and the elderly, could address usability concerns. Moreover, it has also been observed that patients with asthma who were prescribed a single combination inhaler in place of separate inhaled corticosteroid controller and salbutamol reliever therapy were significantly more likely to achieve asthma control compared with those using mixed devices, such as a breath-actuated inhaler for the controller and a pMDI for the reliever.^32^ When patients are switched between inhaler types, it is critical to ensure that proper inhaler techniques are applied, this can include approaches such as the teach-back method and expectation setting regarding the sensory experience of using different inhalers. Whenever possible, the same device for both controller and reliever therapy should be recommended when initiating inhaler therapy, this is also reflected in the most recent GINA guidelines.^28 32^

Recyclability and disposal options present further challenges. Few patients report returning inhalers to pharmacies, and many prescribers are unaware of inhaler disposal or recycling pathways, where they exist.^13 21^ Implementing and promoting proper disposal programmes for pMDIs may reduce their environmental impact, with pharmacists and healthcare providers leading the education of patients on the importance of returning used inhalers for proper disposal.^33^ It is likely that physician leadership in discussing the benefits of recycling inhalers with patients alongside advocacy for system-level changes to facilitate recycling programmes could increase the frequency of inhaler recycling, although further research is required to evaluate this. Legislation requiring manufacturers to contribute handling fees or organise recycling programmes that provide a return incentive has been demonstrated to be successful in other sectors^34^ and may help address system-level changes that are beyond the control of prescribers. Such initiatives that make proper disposal routines seamless and incentivise recycling could also hold promise for reducing the overall environmental footprint of inhalers.

Finally, the transition from CFCs to HFAs underscores the potential for large-scale change when media, policy and clinical practice align.^4^,^6^ In that earlier era, widespread public awareness and multinational agreements ensured patients and clinicians understood both the urgency and the rationale for adopting new inhaler propellants.^4^,^6^ Replicating that success in the shift to DPIs would require similarly coordinated efforts, including clear governmental directives, industry-level investment in inhaler innovation and robust educational campaigns.^4^,^6^ Such efforts could not only alleviate clinical hesitations regarding efficacy and cost but also empower patients with tangible options for reducing their individual carbon footprint.

By integrating models of eco‐ethical leadership and the planetary healthcare framework, healthcare systems can overcome both attitudinal and systemic barriers.^23 24^ Future research should evaluate whether targeted educational interventions (eg, clinical decision tools or dedicated training modules) and standardised prescribing protocols can effectively overcome reluctance and knowledge gaps. Additionally, longitudinal studies comparing clinical outcomes, overall costs and patient satisfaction across inhaler types would provide more definitive evidence for or against transitioning to DPIs on a larger scale. By integrating education, cost considerations and clear organisational policies, healthcare systems may more successfully bridge the gap between willingness and action in sustainable inhaler prescribing.

### Limitations

This review is limited by the small number of included studies (n=4), which may not fully capture the diversity of perspectives and practices globally. The article written by Goh *et al* was published in 1998, this may limit its contextual relevance in 2025, while these factors have been detailed in the Results section, the inclusion of this study provides valuable insight into parallels between the shift away from CFCs and the shift away from pMDIs. The risk of bias assessment using the NOS revealed generally low levels of bias in the included studies. However, some methodological limitations deserve attention. For instance, the reliance on self-reported data introduces reporting bias, or socially desirable responses, particularly concerning attitudes and prescribing patterns. The sample sizes of individual studies were also modest, and the results may be subjected to selection bias, as participants inclined towards environmentally conscious practices might have been over-represented.^16 21^ This review focused mainly on DPIs and pMDIs, while these are the two most commonly prescribed types of inhalers, soft mist inhalers are another alternative to pMDIs that do not require propellant. While these inhalers contribute to more sustainable prescribing, they are less commonly prescribed, likely due to standardised guidelines such as GINA, which now recommend budesonide-formoterol as the first-line treatment in patients over 12 years of age, which is most commonly formulated as a DPI. Over time, as the inhaler market continues to develop, this is an area for future research. Finally, the heterogeneity of study designs precluded meta-analysis, limiting the ability to quantify effect sizes or draw more robust conclusions.

## Conclusion

The transition to environmentally sustainable inhaler practices is a crucial component of reducing the healthcare sector’s carbon footprint. Despite growing recognition of the need for sustainable healthcare practices, pMDIs remain the dominant choice, largely due to entrenched prescribing habits, perceived user-friendliness and patient familiarity. However, healthcare leaders are uniquely positioned to address the dual challenges of improving respiratory health and mitigating environmental impact. This review’s findings suggest that patient and prescriber willingness to switch to DPIs is malleable and could be enhanced. Addressing knowledge gaps, providing financial incentives, improving accessibility and enhancing usability could promote the adoption of DPIs and other sustainable inhalers. By embracing eco‐ethical leadership and the planetary healthcare framework, leaders can align patient concerns and clinical efficacy with sustainability goals.^23 24^ This also requires a concerted leadership effort from providers, policymakers and industry stakeholders. Future research and policy initiatives should focus on understanding global variations in practices, the effectiveness of interventions and long-term outcomes of sustainability-focused strategies in respiratory care to facilitate a shift towards sustainable respiratory care practices, ultimately aligning healthcare with broader principles of planetary health.

## Supplementary material

10.1136/leader-2025-001257 online supplemental file 1

## Data Availability

All data relevant to the study are included in the article or uploaded as supplementary information.
