# Supplementary material for: Clearing the air: a systematic review on leadership challenges with sustainable inhaler prescribing
Source: BMJ Lead. 2025 Aug 6;9(4):e001257. doi: 10.1136/leader-2025-001257 (PMC12772584; doi:10.1136/leader-2025-001257)
Supplement: online supplemental file 1 [file leader-9-4-s001.pdf]

Appendix 1

EMBASE via Elsevier **167**

Results

#1 'general practitioner'/exp OR 'pulmonologist'/exp OR 'physician'/exp

#2 'gps':ti,ab OR 'teenager*':ti,ab OR 'doctors':ti,ab OR 'respiratory':ti,ab OR 'family doctor':ti,ab OR 'family physician':ti,ab OR 'family physicians':ti,ab OR 'family practitioner':ti,ab OR 'general physician':ti,ab OR 'general practice physician':ti,ab OR 'general practitioners':ti,ab OR 'gp (general practitioner)':ti,ab OR 'physicians, family':ti,ab OR 'physicians, primary care':ti,ab OR 'practitioner, general':ti,ab OR 'primary care doctor':ti,ab OR 'primary care physician':ti,ab OR 'primary care physicians':ti,ab OR 'general practitioner':ti,ab OR 'pneumologist':ti,ab OR 'pneumologists':ti,ab OR 'pulmonologists':ti,ab OR 'doctor':ti,ab OR 'medical doctor':ti,ab OR 'medical practitioner':ti,ab OR 'physician associate':ti,ab OR 'practitioner':ti,ab OR 'private physician':ti,ab

#3 #1 OR #2

#4 'metered dose inhaler'/exp OR 'breath-actuated inhaler'/exp OR 'dry powder inhaler'/exp OR 'soft mist inhaler'/exp

#5 'BronchoAir':ti,ab OR 'Calidose':ti,ab OR 'Easi-Breathe':ti,ab OR 'Evohaler':ti,ab OR 'inhaler, metered dose':ti,ab OR 'inhalers, metered dose':ti,ab OR 'K-Haler':ti,ab OR 'MDI (inhalers)':ti,ab OR 'Medihaler':ti,ab OR 'Portex ACE':ti,ab OR 'PulmoSpheres':ti,ab OR 'Redihaler':ti,ab OR 'SmartMist':ti,ab OR 'turbuhaler':ti,ab OR 'Twisthaler':ti,ab OR 'Xcelovent':ti,ab OR '3M Autohaler':ti,ab OR 'Aerodose':ti,ab OR 'AKITA (breath-actuated inhaler)':ti,ab OR 'Autohaler':ti,ab OR 'breath-activated inhaler':ti,ab OR 'Accuhaler':ti,ab OR 'Aeorolizer':ti,ab OR 'Aerohaler':ti,ab OR 'Aerohalor':ti,ab OR 'Aerolizer':ti,ab OR 'Airmax':ti,ab OR 'Aspirair':ti,ab OR 'Auto-Jethaler':ti,ab OR 'Axahaler':ti,ab OR 'BREEZHALER':ti,ab OR 'Certihaler':ti,ab OR 'Clickhaler':ti,ab OR 'Cyclohaler':ti,ab OR 'Diskhaler':ti,ab OR 'Diskus':ti,ab OR 'Diskus inhaler':ti,ab OR 'Dreamboat (dry powder inhaler)':ti,ab OR 'dry powder inhalers':ti,ab OR 'Easyhaler':ti,ab OR 'Ellipta (dry powder inhaler)':ti,ab OR 'Elpenhaler':ti,ab OR 'Flexhaler':ti,ab OR 'FlowCaps':ti,ab OR 'Genuair':ti,ab OR 'HandiHaler':ti,ab OR 'MAGhaler':ti,ab OR 'MedTone':ti,ab OR 'Microdose (dry powder inhaler)':ti,ab OR 'Monodose':ti,ab OR 'Neohaler':ti,ab OR 'NEXThaler':ti,ab OR 'Novolizer':ti,ab OR 'Podhaler':ti,ab OR 'Pressair':ti,ab OR 'Pulvinal (dry powder inhaler)':ti,ab OR 'Pulvinar (dry powder inhaler)':ti,ab OR 'Rotadisk':ti,ab OR 'Rotadisk inhaler':ti,ab OR 'rotahaler':ti,ab OR 'Rotohaler':ti,ab OR 'Skyehaler':ti,ab OR 'Spinhaler':ti,ab OR 'Spiromax':ti,ab OR 'T-326 Inhaler':ti,ab OR 'Taifun':ti,ab OR 'Turbohaler':ti,ab OR 'Turbospin':ti,ab OR 'TwinCaps':ti,ab OR 'Twincer':ti,ab OR 'Ultrahaler':ti,ab OR 'Mystic (soft mist inhaler)':ti,ab OR 'RapidMist':ti,ab OR 'Respimat':ti,ab OR 'mdi':ti,ab OR 'propellant inhalers':ti,ab OR 'pressurized inhalers':ti,ab OR 'aerosol inhalers':ti,ab OR 'dpi':ti,ab OR 'powder inhalers':ti,ab OR 'breath-activated inhalers':ti,ab

#6 #4 OR #5

#7 'environmental impact'/exp OR 'environmental sustainability'/exp OR 'environmental protection'/exp OR 'environmental decision making'/exp OR 'pollution control'/exp OR 'global change'/exp

#8 'environmental fate':ti,ab OR 'sustainability, environmental':ti,ab OR 'conservation of natural resources':ti,ab OR 'conservation, environmental':ti,ab OR 'conservation, natural

resource':ti,ab OR 'conservation, nature':ti,ab OR 'environmental conservation':ti,ab OR 'natural resource conservation':ti,ab OR 'nature conservation':ti,ab OR 'nature preservation':ti,ab OR 'protection, environmental':ti,ab OR 'environmental decisioning':ti,ab OR 'sustainable healthcare practices':ti,ab OR 'eco-friendly healthcare':ti,ab OR 'green healthcare':ti,ab OR 'environmentally sustainable healthcare':ti,ab OR 'sustainable medical practices':ti,ab OR 'environmentally responsible healthcare':ti,ab OR 'sustainable health practices':ti,ab OR 'environmentally friendly healthcare':ti,ab OR 'sustainable medical care':ti,ab OR 'eco-conscious healthcare':ti,ab OR 'green medical practices':ti,ab OR 'sustainable health care':ti,ab OR 'Environmental Health':ti,ab OR 'Environmental Pollutants':ti,ab OR 'Environmental Pollution':ti,ab OR 'Air Pollutants':ti,ab OR 'Global Warming':ti,ab OR 'Global Health':ti,ab OR 'Climate Change':ti,ab OR 'Change Management':ti,ab OR 'Environmental Policy':ti,ab OR 'chemical micropollutant':ti,ab OR 'environment pollutant':ti,ab OR 'environmental pollutants':ti,ab OR 'pollutant agent':ti,ab OR 'radioactive pollutants':ti,ab OR 'aerial pollution':ti,ab OR 'aerogenic pollution':ti,ab OR 'air contamination':ti,ab OR 'air pollutioning':ti,ab OR 'air-borne pollution':ti,ab OR 'airborne pollution':ti,ab OR 'atmosphere pollution':ti,ab OR 'atmospheric pollution':ti,ab OR 'polluted air':ti,ab OR 'polluted atmosphere':ti,ab OR 'pollution, air':ti,ab OR 'air pollution':ti,ab OR 'air pollutants, environmental':ti,ab OR 'air pollutants, occupational':ti,ab OR 'air pollutants, radioactive':ti,ab OR 'pollutant, air':ti,ab OR 'air pollutant':ti,ab OR 'environment contamination':ti,ab OR 'environment pollution':ti,ab OR 'pollution, environmental':ti,ab OR 'pollution':ti,ab OR 'pollutant':ti,ab OR 'effect, greenhouse':ti,ab OR 'warming, global':ti,ab OR 'greenhouse effect':ti,ab OR 'world health':ti,ab OR 'world-wide health':ti,ab OR 'worldwide health':ti,ab OR 'climate sensitivity':ti,ab OR 'climate variability':ti,ab OR 'climatic change':ti,ab OR 'greenhouse gases':ti,ab OR 'greenhouse gas':ti,ab

#9 #7 OR #8

#10 #3 AND #6 AND #9

Medline via EBSCO 185 Results

1. (MH "General Practitioners") OR (MH "Physicians+") OR (MH "Pulmonary Medicine") OR (MH "Pulmonologists") OR (MH "Child+") OR (MH "Adolescent")
2. AB ("GPs" OR "Doctors" OR "Respiratory" OR "family doctor" OR "family physician" OR "family physicians" OR "family practitioner" OR "general physician" OR "general practice physician" OR "GP (general practitioner)" OR "physicians, family" OR "physicians, primary care" OR "practitioner, general" OR "primary care doctor" OR "primary care physician" OR "primary care physicians" OR "pneumologist" OR "pneumologists" OR "doctor" OR "medical doctor" OR "medical practitioner" OR "physician associate" OR "practitioner" OR "private physician") OR TI ("GPs" OR "Doctors" OR "Respiratory" OR "family doctor" OR "family physician" OR "family physicians" OR "family practitioner" OR "general physician" OR "general practice physician" OR "GP (general practitioner)" OR "physicians, family" OR "physicians, primary care" OR "practitioner, general" OR "primary care doctor" OR "primary care physician" OR "primary care physicians" OR

"pneumologist" OR "pneumologists" OR "doctor" OR "medical doctor" OR "medical practitioner" OR "physician associate" OR "practitioner" OR "private physician")

3. S1 OR S2
4. (MH ”Metered Dose Inhalers+") OR (MH "Dry Powder Inhalers")
5. AB ("breath-actuated inhaler" OR "soft mist inhaler" OR "BronchoAir" OR "Calidose" OR "Easi-Breathe" OR "Evohaler" OR "inhaler, metered dose" OR "inhalers, metered dose" OR "K-Haler" OR "MDI (inhalers)" OR "Medihaler" OR "Portex ACE" OR "PulmoSpheres" OR "Redihaler" OR "SmartMist" OR "turbuhaler" OR "Twisthaler" OR "Xcelovent" OR "3M Autohaler" OR "Aerodose" OR "AKITA (breath-actuated inhaler)" OR "Autohaler" OR "breath-activated inhaler" OR "Accuhaler" OR "Aeorolizer" OR "Aerohaler" OR "Aerohalor" OR "Aerolizer" OR "Airmax" OR "Aspirair" OR "Auto-Jethaler" OR "Axahaler" OR "BREEZHALER" OR "Certihaler" OR "Clickhaler" OR "Cyclohaler" OR "Diskhaler" OR "Diskus" OR "Diskus inhaler" OR "Easyhaler" OR "Elpenhaler" OR "Flexhaler" OR "FlowCaps" OR "Genuair" OR "HandiHaler" OR "MAGhaler" OR "MedTone" OR "Monodose" OR "Neohaler" OR "NEXThaler" OR "Novolizer" OR "Podhaler" OR "Pressair" OR "Rotadisk" OR "Rotadisk inhaler" OR "rotahaler" OR "Rotohaler" OR "Skyehaler" OR "Spinhaler" OR "Spiromax" OR "T-326 Inhaler" OR "Taifun" OR "Turbohaler" OR "Turbospin" OR "TwinCaps" OR "Twincer" OR "Ultrahaler" OR "Mystic (soft mist inhaler)" OR "RapidMist" OR "Respimat" OR "mdi" OR "propellant inhalers" OR "pressurized inhalers" OR "aerosol inhalers" OR "dpi" OR "powder inhalers" OR "breath-activated inhalers") OR TI ("breath-actuated inhaler" OR "soft mist inhaler" OR "BronchoAir" OR "Calidose" OR "Easi-Breathe" OR "Evohaler" OR "inhaler, metered dose" OR "inhalers, metered dose" OR "K-Haler" OR "MDI (inhalers)" OR "Medihaler" OR "Portex ACE" OR "PulmoSpheres" OR "Redihaler" OR "SmartMist" OR "turbuhaler" OR "Twisthaler" OR "Xcelovent" OR "3M Autohaler" OR "Aerodose" OR "AKITA (breath-actuated inhaler)" OR "Autohaler" OR "breath-activated inhaler" OR "Accuhaler" OR "Aeorolizer" OR "Aerohaler" OR "Aerohalor" OR "Aerolizer" OR "Airmax" OR "Aspirair" OR "Auto-Jethaler" OR "Axahaler" OR "BREEZHALER" OR "Certihaler" OR "Clickhaler" OR "Cyclohaler" OR "Diskhaler" OR "Diskus" OR "Diskus inhaler" OR "Easyhaler" OR "Elpenhaler" OR "Flexhaler" OR "FlowCaps" OR "Genuair" OR "HandiHaler" OR "MAGhaler" OR "MedTone" OR "Monodose" OR "Neohaler" OR "NEXThaler" OR "Novolizer" OR "Podhaler" OR "Pressair" OR "Rotadisk" OR "Rotadisk inhaler" OR "rotahaler" OR "Rotohaler" OR "Skyehaler" OR "Spinhaler" OR "Spiromax" OR "T-326 Inhaler" OR "Taifun" OR "Turbohaler" OR "Turbospin" OR "TwinCaps" OR "Twincer" OR "Ultrahaler" OR "Mystic (soft mist inhaler)" OR "RapidMist" OR "Respimat" OR "mdi" OR "propellant inhalers" OR "pressurized inhalers" OR "aerosol inhalers" OR "dpi" OR "powder inhalers" OR "breath-activated inhalers")
6. S4 OR S5
7. (MH "Environmental Health+") OR (MH "Environmental Pollutants+") OR (MH "Environmental Pollution+") OR (MH "Air Pollutants+") OR (MH "Global Warming") OR (MH "Global Health") OR (MH "Climate Change+") OR (MH

"Change Management") OR (MH "Environmental Policy") OR (MH "Air Pollution+") OR (MH "Greenhouse Gases")

8. AB ("environmental impact" OR "environmental sustainability" OR "environmental protection" OR "environmental decision making" OR "pollution control" OR "global change" OR "environmental fate" OR "sustainability, environmental" OR "conservation of natural resources" OR "conservation, environmental" OR "conservation, natural resource" OR "conservation, nature" OR "environmental conservation" OR "natural resource conservation" OR "nature conservation" OR "nature preservation" OR "protection, environmental" OR "environmental decisioning" OR "sustainable healthcare practices" OR "eco-friendly healthcare" OR "green healthcare" OR "environmentally sustainable healthcare" OR "sustainable medical practices" OR "environmentally responsible healthcare" OR "sustainable health practices" OR "environmentally friendly healthcare" OR "sustainable medical care" OR "eco-conscious healthcare" OR "green medical practices" OR "sustainable health care" OR "chemical micropollutant" OR "environment pollutant" OR "pollutant agent" OR "radioactive pollutants" OR "aerial pollution" OR "aerogenic pollution" OR "air contamination" OR "air pollutioning" OR "air-borne pollution" OR "airborne pollution" OR "atmosphere pollution" OR "atmospheric pollution" OR "polluted air" OR "polluted atmosphere" OR "pollution, air" OR "air pollutants, environmental" OR "air pollutants, occupational" OR "air pollutants, radioactive" OR "pollutant, air" OR "air pollutant" OR "environment contamination" OR "pollution, environmental" OR "pollution" OR "pollutant" OR "effect, greenhouse" OR "warming, global" OR "greenhouse effect" OR "world health" OR "world-wide health" OR "worldwide health" OR "climate sensitivity" OR "climate variability" OR "climatic change" OR "greenhouse gas") OR TI ("environmental impact" OR "environmental sustainability" OR "environmental protection" OR "environmental decision making" OR "pollution control" OR "global change" OR "environmental fate" OR "sustainability, environmental" OR "conservation of natural resources" OR "conservation, environmental" OR "conservation, natural resource" OR "conservation, nature" OR "environmental conservation" OR "natural resource conservation" OR "nature conservation" OR "nature preservation" OR "protection, environmental" OR "environmental decisioning" OR "sustainable healthcare practices" OR "eco-friendly healthcare" OR "green healthcare" OR "environmentally sustainable healthcare" OR "sustainable medical practices" OR "environmentally responsible healthcare" OR "sustainable health practices" OR "environmentally friendly healthcare" OR "sustainable medical care" OR "eco-conscious healthcare" OR "green medical practices" OR "sustainable health care" OR "chemical micropollutant" OR "environment pollutant" OR "pollutant agent" OR "radioactive pollutants" OR "aerial pollution" OR "aerogenic pollution" OR "air contamination" OR "air pollutioning" OR "air-borne pollution" OR "airborne pollution" OR "atmosphere pollution" OR "atmospheric pollution" OR "polluted air" OR "polluted atmosphere" OR "pollution, air" OR "air pollutants, environmental" OR "air pollutants, occupational" OR "air pollutants, radioactive" OR "pollutant, air" OR "air pollutant" OR "environment contamination" OR "pollution, environmental" OR "pollution" OR "pollutant" OR

"effect, greenhouse" OR "warming, global" OR "greenhouse effect" OR "world health" OR "world-wide health" OR "worldwide health" OR "climate sensitivity" OR "climate variability" OR "climatic change" OR "greenhouse gas")
9. S7 OR S8
10. S3 AND S6 AND S9

Web of Science **75 Results**

1. TS=("general practitioner" OR "pulmonologist" OR "physician" OR "gps" OR "teenager" OR "doctors" OR "respiratory" OR "family doctor" OR "family physician" OR "family physicians" OR "family practitioner" OR "general physician" OR "general practice physician" OR "general practitioners" OR "gp (general practitioner)" OR "physicians, family" OR "physicians, primary care" OR "practitioner, general" OR "primary care doctor" OR "primary care physician" OR "primary care physicians" OR "pneumologist" OR "pneumologists" OR "pulmonologists" OR "doctor" OR "medical doctor" OR "medical practitioner" OR "physician associate" OR "practitioner" OR "private physician")
2. TS=("metered dose inhaler" OR "breath-actuated inhaler" OR "dry powder inhaler" OR "soft mist inhaler" OR "BronchoAir" OR "Calidose" OR "Easi-Breathe" OR "Evohaler" OR "inhaler, metered dose" OR "inhalers, metered dose" OR "K-Haler" OR "MDI (inhalers)" OR "Medihaler" OR "Portex ACE" OR "PulmoSpheres" OR "Redihaler" OR "SmartMist" OR "turbuhaler" OR "Twisthaler" OR "Xcelovent" OR "3M Autohaler" OR "Aerodose" OR "AKITA (breath-actuated inhaler)" OR "Autohaler" OR "breath-activated inhaler" OR "Accuhaler" OR "Aeorolizer" OR "Aerohaler" OR "Aerohalor" OR "Aerolizer" OR "Airmax" OR "Aspirair" OR "Auto-Jethaler" OR "Axahaler" OR "BREEZHALER" OR "Certihaler" OR "Clickhaler" OR "Cyclohaler" OR "Diskhaler" OR "Diskus" OR "Diskus inhaler" OR "Dreamboat" OR "Easyhaler" OR "Ellipta" OR "Elpenhaler" OR "Flexhaler" OR "FlowCaps" OR "Genuair" OR "HandiHaler" OR "MAGhaler" OR "MedTone" OR "Microdose" OR "Monodose" OR "Neohaler" OR "NEXThaler" OR "Novolizer" OR "Podhaler" OR "Pressair" OR "Pulvinal" OR "Pulvinar" OR "Rotadisk" OR "Rotadisk inhaler" OR "rotahaler" OR "Rotohaler" OR "Skyehaler" OR "Spinhaler" OR "Spiromax" OR "T-326 Inhaler" OR "Taifun" OR "Turbohaler" OR "Turbospin" OR "TwinCaps" OR "Twincer" OR "Ultrahaler" OR "Mystic" OR "RapidMist" OR "Respimat" OR "mdi" OR "propellant inhalers" OR "pressurized inhalers" OR "aerosol inhalers" OR "dpi" OR "powder inhalers" OR "breath-activated inhalers")
3. TS=("environmental impact" OR "environmental sustainability" OR "environmental protection" OR "environmental decision making" OR "pollution control" OR "global change" OR "environmental fate" OR "sustainability, environmental" OR "conservation of natural resources" OR "conservation, environmental" OR "conservation, natural resource" OR "conservation, nature" OR "environmental conservation" OR "natural resource conservation" OR "nature conservation" OR "nature preservation" OR "protection, environmental" OR "environmental decisioning" OR "sustainable healthcare practices" OR "eco-friendly healthcare" OR "green healthcare" OR "environmentally sustainable healthcare" OR "sustainable

medical practices" OR "environmentally responsible healthcare" OR "sustainable health practices" OR "environmentally friendly healthcare" OR "sustainable medical care" OR "eco-conscious healthcare" OR "green medical practices" OR "sustainable health care" OR "Environmental Health" OR "Environmental Pollutants" OR "Environmental Pollution" OR "Air Pollutants" OR "Global Warming" OR "Global Health" OR "Climate Change" OR "Change Management" OR "Environmental Policy" OR "chemical micropollutant" OR "environment pollutant" OR "pollutant agent" OR "radioactive pollutants" OR "aerial pollution" OR "aerogenic pollution" OR "air contamination" OR "air pollutioning" OR "air-borne pollution" OR "airborne pollution" OR "atmosphere pollution" OR "atmospheric pollution" OR "polluted air" OR "polluted atmosphere" OR "pollution, air" OR "air pollutants, environmental" OR "air pollutants, occupational" OR "air pollutants, radioactive" OR "pollutant, air" OR "air pollutant" OR "environment contamination" OR "pollution, environmental" OR "pollution" OR "pollutant" OR "effect, greenhouse" OR "warming, global" OR "greenhouse effect" OR "world health" OR "world-wide health" OR "worldwide health" OR "climate sensitivity" OR "climate variability" OR "climatic change" OR "greenhouse gases" OR "greenhouse gas")
4. #1 AND #2 AND #3

Gray Literature: Google Scholar 5,700 results
(Patient* OR Physician*) AND (Metered Dose Inhalers OR Dry Powder Inhalers) AND (Environment OR sustainabl*) AND (attitude* OR preference*)

Appendix 2: Eligibility Criteria

Population *(P)*
Inclusion Criteria:
- Studies involving physicians.
- Studies focusing on patients.

Exclusion Criteria:
- Studies focusing on healthcare providers other than GPs, Pediatricians, Pulmonologists, or Primary Care Providers (e.g., nurses, pharmacists).

Intervention *(I)*
Inclusion Criteria:
- Studies examining the use, awareness, and attitudes towards Metered Dose Inhalers (MDIs).

Exclusion Criteria:
- Studies that do not include any intervention related to inhaler prescription practices.

Comparison *(C)*
Inclusion Criteria:
- Studies comparing MDIs with Dry Powder Inhalers (DPIs).
- Studies that include DPIs as a comparison group to MDIs.

Exclusion Criteria:
- Studies that focus exclusively on one type of inhaler without a comparative analysis.

Outcomes *(O)*

Inclusion Criteria:
- Studies reporting on patient's or physicians' awareness, attitudes, and perceptions regarding the environmental impact of inhalers.
- Studies discussing the factors influencing prescribing practices, including barriers and facilitators to adopting DPIs.
- Studies examining the knowledge and attitudes towards sustainable healthcare practices.

Exclusion Criteria:
- Studies lacking relevant outcomes pertaining to sustainable healthcare practices or environmental sustainability.

Additional Criteria

Study Design*:*

- Inclusion: Qualitative and quantitative studies, including surveys, poster abstracts, interviews, observational studies, letters to the editor, and cross-sectional studies.

- Exclusion: Case reports, editorials, and commentaries. Studies with insufficient methodological quality or incomplete data.

Study	Selection	Comparability	Outcome
Woodall / 2023	★★★★	★★	★
Walpole / 2021	★★★★	★★	★
Goh / 1998	★★★★	★★	★
Quantz / 2023	★★★★	★★	★

Quality Assessment Table

NOS Category
Selection
Comparability
Outcome

Author Affiliations and Bias Assessment

Author
Matthew J Woo
John Ma
Kate Emett
Amelia PE Ham
Katie Knowles
Tom Hyunwoo
Wilson Mitchell
Wennarator Ira
Letoe Renee T
John D Dockert
Robert J Hanc

Criteria	Score
Representativeness of the exposed cohort	★
Selection of the non-exposed cohort	★
Ascertainment of exposure	★
Demonstration that outcome was not present at start of study	★
Comparability of cohorts on the basis of the design or analysis	★★
Assessment of outcome	★
Was follow-up long enough for outcomes to occur	Ø
Adequacy of follow-up of cohorts	Ø

Affiliations	Bias
Trainee Intern, Dept. of Preventive & Social Medicine, Univ. of Otago	No ider
Trainee Intern, Dept. of Preventive & Social Medicine, Univ. of Otago	No ider
Trainee Intern, Dept. of Preventive & Social Medicine, Univ. of Otago	No ider
Trainee Intern, Dept. of Preventive & Social Medicine, Univ. of Otago	No ider
Trainee Intern, Dept. of Preventive & Social Medicine, Univ. of Otago	No ider
Trainee Intern, Dept. of Preventive & Social Medicine, Univ. of Otago	No ider
Trainee Intern, Dept. of Preventive & Social Medicine, Univ. of Otago	No ider
Trainee Intern, Dept. of Preventive & Social Medicine, Univ. of Otago	No ider
Trainee Intern, Dept. of Preventive & Social Medicine, Univ. of Otago	No ider
Associate Professor, Dept. of Preventive & Social Medicine, Univ. of (	No ider
Professor, Dept. of Preventive & Social Medicine, Univ. of Otago	No ider

Justification

Patients and practitioners from primary and secondary care settings in Dunedin and Invercargill.

Comparisons between users of pMDIs and DPIs were made.

Exposure assessed via structured questionnaires.

Participants were surveyed about current inhaler use and attitudes, ensuring pre-exposure status.

Controlled for age, gender, and education level in the analysis.

Outcomes assessed through validated surveys and statistical analysis.

Study was cross-sectional; no long-term follow-up.

Not applicable due to the cross-sectional nature.

ntified bias. The author is a trainee intern with no disclosed conflicts of interest.

ntified bias. The author is a trainee intern with no disclosed conflicts of interest.

ntified bias. The author is a trainee intern with no disclosed conflicts of interest.

ntified bias. The author is a trainee intern with no disclosed conflicts of interest.

ntified bias. The author is a trainee intern with no disclosed conflicts of interest.

ntified bias. The author is a trainee intern with no disclosed conflicts of interest.

ntified bias. The author is a trainee intern with no disclosed conflicts of interest.

ntified bias. The author is a trainee intern with no disclosed conflicts of interest.

ntified bias. The author is a trainee intern with no disclosed conflicts of interest.

ntified bias. The author is an associate professor with no disclosed conflicts of interest.

ntified bias. The author is a professor with no disclosed conflicts of interest.

Quality Assessment Table

NOS Category
Selection
Comparability
Outcome

Author Affiliations and Bias Assessment

Author
Sarah C Walpo
A Katherine Sm
Joseph McElva
Jill Taylor
Simon Doe
Hilary Tedd

Criteria	Score
Representativeness of the exposed cohort	★
Selection of the non-exposed cohort	★
Ascertainment of exposure	★
Demonstration that outcome was not present at start of study	★
Comparability of cohorts on the basis of the design or analysis	★★
Assessment of outcome	★
Was follow-up long enough for outcomes to occur	Ø
Adequacy of follow-up of cohorts	Ø

Affiliations	Bias
Dept. of Infectious Diseases, The Newcastle upon Tyne Hospitals NH	No ider
Trust Doctor, The Newcastle upon Tyne Hospitals NHS FT	No ider
Medical Student, Newcastle University	No ider
Respiratory Pharmacist, The Newcastle upon Tyne Hospitals NHS FT	No ider
Consultant in Respiratory Medicine, The Newcastle upon Tyne Hospit	No ider
Consultant in Respiratory Medicine, The Newcastle upon Tyne Hospit	No ider

Justification

Included prescribers from various specialties within one NHS trust, ensuring a diverse sample.

Comparisons between non-respiratory specialties and respiratory specialists

Exposure assessed via structured surveys distributed electronically and in person.

Survey focused on current knowledge and practices, ensuring pre-exposure status.

Controlled for professional role and previous experience in respiratory settings.

Outcomes assessed through validated surveys and statistical analysis.

Study was cross-sectional; no long-term follow-up.

Not applicable due to the cross-sectional nature.

ntified bias. The author is affiliated with a reputable institution with no disclosed conflicts.

ntified bias. The author is affiliated with a reputable institution with no disclosed conflicts.

ntified bias. The author is a medical student with no disclosed conflicts.

ntified bias. The author is affiliated with a reputable institution with no disclosed conflicts.

ntified bias. The author is a consultant with disclosed conflicts related to speaker fees.

ntified bias. The author is a consultant with no disclosed conflicts.

Quality Assessment Table

NOS Category	Criteria
Selection	Representativeness of the exposed cohort
	Selection of the non-exposed cohort
	Ascertainment of exposure
	Demonstration that outcome was not present at start of stud
Comparability	Comparability of cohorts on the basis of the design or analys
Outcome	Assessment of outcome
	Was follow-up long enough for outcomes to occur
	Adequacy of follow-up of cohorts

Author Affiliations and Bias Assessment

Author	Affiliations
S.Y. Goh	Department of Pediatrics, National University of Singapore, S
S. Arulanandar	Department of Pediatrics, National University of Singapore, S
C.L. Ho	Department of Pediatrics, National University of Singapore, S
L. Zhang	Department of Pediatrics, National University of Singapore, S
D.Y.T. Goh	Department of Pediatrics, National University of Singapore, S
F.T. Chew	Department of Pediatrics, National University of Singapore, S

Score	Justification
★	Parents of pediatric patients from a hospital setting, representing a relevant population.
★	Controls were selected from the same population without prior knowledge of CFC-free inhale
★	Exposure assessed via structured interviews.
★	Ensured pre-exposure status by focusing on current knowledge and practices.
★★	Controlled for demographic factors such as age, education, and socioeconomic status.
★	Outcomes assessed through validated interviews and statistical analysis.
Ø	Study was cross-sectional; no long-term follow-up.
Ø	Not applicable due to the cross-sectional nature.

Bias
None identified
None identified
None identified
None identified
None identified
None identified

rs.

Quality Assessment Table

NOS Category
Selection
Comparability
Outcome

Author Affiliations and Bias Assessment

Author
Darryl Quantz
Gigi Y.C. Wong
Kevin Liang

Criteria	Score
Representativeness of the exposed cohort	★
Selection of the non-exposed cohort	★
Ascertainment of exposure	★
Demonstration that outcome was not present at start of study	★
Comparability of cohorts on the basis of the design or analysis	★★
Assessment of outcome	★
Was follow-up long enough for outcomes to occur	Ø
Adequacy of follow-up of cohorts	Ø

Affiliations	Bias
Fraser Health Authority, Surrey, BC	No ider
Lower Mainland Pharmacy Services, Surrey, BC	No ider
Fraser Health Authority, Surrey, BC	No ider

Justification

Participants were residents from the most populous health region in British Columbia, representing a diverse sam[illegible]
The study compared those aware and unaware of the environmental impact of inhalers.
Exposure assessed via structured online surveys.
Survey focused on current inhaler use and attitudes, ensuring pre-exposure status.
Controlled for age, gender, and primary diagnosis (asthma or COPD) in the analysis.
Outcomes assessed through validated surveys and statistical analysis.
Study was cross-sectional; no long-term follow-up.
Not applicable due to the cross-sectional nature.

[illegible]ntified bias. The author is affiliated with a reputable health authority with no conflicts.
[illegible]ntified bias. The author is affiliated with a reputable pharmacy service with no conflicts.
[illegible]ntified bias. The author is affiliated with a reputable health authority with no conflicts.

ple.
